# Structural Racism, Mass Incarceration, and Racial and Ethnic Disparities in Severe Maternal Morbidity

**DOI:** 10.1001/jamanetworkopen.2023.53626

**Published:** 2024-01-26

**Authors:** Elleni M. Hailu, Corinne A. Riddell, Patrick T. Bradshaw, Jennifer Ahern, Suzan L. Carmichael, Mahasin S. Mujahid

**Affiliations:** 1 Division of Epidemiology, School of Public Health, University of California, Berkeley; 2 Division of Biostatistics, School of Public Health, University of California, Berkeley; 3 Division of Neonatal and Developmental Medicine, Department of Pediatrics, Stanford University, Palo Alto, California; 4 Division of Maternal-Fetal Medicine and Obstetrics, School of Medicine, Stanford University, Palo Alto, California

## Abstract

**Importance:**

Racial and ethnic inequities in the criminal-legal system are an important manifestation of structural racism. However, how these inequities may influence the risk of severe maternal morbidity (SMM) and its persistent racial and ethnic disparities remains underinvestigated.

**Objective:**

To examine the association between county-level inequity in jail incarceration rates comparing Black and White individuals and SMM risk in California.

**Design, Setting, and Participants:**

This population-based cross-sectional study used state-wide data from California on all live hospital births at 20 weeks of gestation or later from January 1, 1997, to December 31, 2018. Data were obtained from hospital discharge and vital statistics records, which were linked with publicly available county-level data. Data analysis was performed from January 2022 to February 2023.

**Exposure:**

Jail incarceration inequity was determined from the ratio of jail incarceration rates of Black individuals to those of White individuals and was categorized as tertile 1 (low), tertile 2 (moderate), tertile 3 (high), with mean cutoffs across all years of 0 to 2.99, 3.00 to 5.22, and greater than 5.22, respectively.

**Main Outcome and Measures:**

This study used race- and ethnicity-stratified mixed-effects logistic regression models with birthing people nested within counties and adjusted for individual- and county-level characteristics to estimate the odds of non–blood transfusion SMM (NT SMM) and SMM including blood transfusion–only cases (SMM; as defined by the Centers for Disease Control and Prevention SMM index) associated with tertiles of incarceration inequity.

**Results:**

This study included 10 200 692 births (0.4% American Indian or Alaska Native, 13.4% Asian or Pacific Islander, 5.8% Black, 50.8% Hispanic or Latinx, 29.6% White, and 0.1% multiracial or other [individuals who self-identified with ≥2 racial groups and those who self-identified as “other” race or ethnicity]). In fully adjusted models, residing in counties with high jail incarceration inequity (tertile 3) was associated with higher odds of SMM for Black (odds ratio [OR], 1.14; 95% CI, 1.01-1.29 for NT SMM; OR, 1.20, 95% CI, 1.01-1.42 for SMM), Hispanic or Latinx (OR, 1.24; 95% CI, 1.14-1.34 for NT SMM; OR, 1.20; 95% CI, 1.14-1.27 for SMM), and White (OR, 1.02; 95% CI, 0.93-1.12 for NT SMM; OR, 1.09; 95% CI, 1.02-1.17 for SMM) birthing people, compared with residing in counties with low inequity (tertile 1).

**Conclusions and Relevance:**

The findings of this study highlight the adverse maternal health consequences of structural racism manifesting via the criminal-legal system and underscore the need for community-based alternatives to inequitable punitive practices.

## Key Points

**Question:**

Is county-level jail incarceration inequity between Black and White individuals, as a manifestation of structural racism, associated with severe maternal morbidity risk?

**Findings:**

In this cross-sectional study of 10 200 692 live hospital births across California between 1997 and 2018, Black and Hispanic or Latinx birthing people residing in counties with high Black-White jail incarceration inequity had increased odds of severe maternal morbidity compared with birthing people residing in low-inequity counties.

**Meaning:**

Structural racism operating within the criminal-legal system may drive racial and ethnic inequities in pregnancy-related complications, indicating the need to transform inequitable institutions in order to improve maternal health outcomes in the US.

## Teaser

This cross-sectional study examines whether individuals residing in counties with the greatest inequity in jail incarceration rates between Black and White individuals have increased risk of severe maternal morbidity.

## Introduction

Each year, severe maternal morbidity (SMM) affects more than 50 000 birthing people in the US.^1^ Severe maternal morbidity is a set of life-threatening physiologic complications and lifesaving procedures occurring during childbirth and post partum that have serious implications for the survival of a person giving birth.^1^ There are persistent racial and ethnic disparities in SMM risk. Black individuals are 2 to 3 times more likely to experience SMM than White individuals.^2,3,4,5^ Furthermore, the US has the highest rates of maternal mortality among high-resource nations, with Black birthing people facing a 3- to 4-fold higher risk of maternal mortality than White individuals. The US is facing a maternal health crisis,^6,7,8,9^ and addressing SMM and its racial and ethnic disparities is critical to improving birthing outcomes across the nation.^10,11^

Because prior work on individual-level risk factors for SMM has not been able to explain racial and ethnic disparities, scholars and professional organizations have called for the need to assess structural determinants of health inequities in relation to SMM.^11,12,13,14^ One such determinant is structural racism—a fundamental cause of racial and ethnic health inequities, defined as a set of historically rooted, intrinsically linked, and mutually reinforcing systems and institutions that work in concert to disenfranchise racially and ethnically marginalized populations.^15,16,17,18,19^ Structural racism perpetuates inequitable policies, practices, and discriminatory social norms that directly and indirectly pattern the distribution of health-promoting material resources, socioeconomic opportunities, and psychosocial stressors and assets, which in turn influence disease risk, including poor birthing outcomes.^15,20,21,22^

The criminal-legal system is one important manifestation of structural racism that continues to disproportionately institutionalize millions of Black and other racially and ethnically marginalized individuals in the US and tear apart countless families and communities.^23,24^ Seemingly race-neutral policies and practices that are fueled by racism and classism and inherently rooted in social and political reactions to the civil rights movement, such as the war on drugs, have led to the imprisonment of countless racially and ethnically marginalized individuals and have made the US a world leader in incarceration.^23,25,26,27,28,29^ As a result of continued underinvestment in health and social services that promote social, emotional, and financial well-being and racist policing and sentencing practices within racially marginalized communities, there are notable racial inequities in the criminal-legal system.^23,28,30,31^ Black individuals have higher incarceration rates than any other racial group.^32^ Hence, they and their communities are more likely to bear the collateral social, emotional, financial, mental, and physical consequences of mass incarceration.^33,34,35^

In addition to the harm it causes to incarcerated people and their families, mass incarceration is known to have ill health effects that permeate communities.^36^ Mass incarceration is conceptualized to influence population health inequities through depleted community socioeconomic and psychosocial resources, severed social ties, and heightened stress.^36,37,38^ Consequently, residing in neighborhoods affected by mass incarceration has been shown to increase the risk of various adverse mental and physical health outcomes.^39,40,41,42,43,44,45,46^ A small but increasing area of empirical work has also begun to document how living in areas with greater racial inequities in incarceration rates may shape adverse birthing outcomes.^43,47,48,49^ However, how the manifestation of structural racism via the criminal-legal system influences SMM risk remains underinvestigated. To our knowledge, only 2 prior studies have examined this relationship and did not detect any associations,^50,51^ highlighting the need for research that further explicates this link.

In this cross-sectional study, we leveraged 21 years of data from California on county-level jail incarceration inequity comparing Black and White individuals as an indicator of structural racism and examined how it may be associated with SMM risk. We hypothesized that individuals residing in counties with high jail incarceration inequity would have increased risk of SMM.

## Methods

### Study Sample

Study data were obtained from the California Department of Health Care Access and Information and included all live births delivered at 20 weeks of gestation or later within the state from January 1, 1997, to December 31, 2018. We kept the first recorded birth for nonsingleton births to avoid duplicates. Birth hospitalization discharge and vital statistics records were linked with county-level jail incarceration data from the Vera Institute of Justice Incarceration Trends data set,^52,53^ the US Census, and American Community Survey (ACS) based on maternal addresses recorded at birth. After removing observations with missing or invalid covariates, the final analytic sample was 10 200 692 births across 57 counties in California (eFigure in Supplement 1). On average, there were 178 960 observations per county throughout the study period (range, 288-2 847 438). This study was approved by the State of California Committee for the Protection of Human Subjects and the institutional review boards of Stanford University and the University of California, Berkeley, which deemed the study exempt from needing to obtain participant informed consent given the nature of the data. This study followed the Strengthening the Reporting of Observational Studies in Epidemiology (STROBE) reporting guideline for cross-sectional studies.

### Study Outcome

To determine the occurrence of SMM during birth hospitalization, we used the Centers for Disease Control and Prevention’s SMM index designed for use in administrative data sets.^1^ This index, which includes a list of 21 life-threatening events and life-saving procedures that occur during childbirth, was constructed using the *International Classification of Diseases, Ninth Revision* (*ICD-9*) and *International Statistical Classification of Diseases and Related Health Problems, Tenth Revision* (*ICD-10*) diagnosis and procedure codes from birth hospital discharge files. Because blood transfusion may overestimate true SMM prevalence given that transfusion volume is unspecified in the *ICD* codes, we consider SMM excluding cases with blood transfusion as their only indicator (non–blood transfusion SMM [NT SMM]) as well as SMM including cases with blood transfusion as their only indicator (SMM).^54,55,56^

### Exposure

Annual county-level jail incarceration rates for the years 1997 to 2018 were obtained from the Vera Institute of Justice Incarceration Trends data set.^53^ This data set compiles incarceration data from the Bureau of Justice Statistics Census of Jails and the Annual Survey of Jails and uses information from National Vital Statistics on county population density (ages 15-64 years) as the denominator to ascertain yearly county-level overall and race- and ethnicity-specific incarceration rates (per 100 000 residents).^52,53^ In other words, these rates are determined based on the proportion of residents of a county who are incarcerated in jails. Racial inequity in jail incarceration rates was defined using the ratio of Black to White jail incarceration rates (race and ethnicity of incarcerated individuals were reported by jail officials). This measure is thought to indicate long-term disinvestment and inequity at the area level that is relevant to the health of all birthing people.^43,47,50,57^ To quantify risk associated with low, moderate, and high exposure to structural racism, which facilitates an easier interpretation, we categorized counties into tertiles of these annual ratios: low inequity (tertile 1 [reference]), moderate inequity (tertile 2), and high inequity (tertile 3). The mean tertile cutoffs across all study years were as follows: tertile 1, 0 to 2.99; tertile 2, 3.00 to 5.22; and tertile 3, greater than 5.22. Each year of exposure data was then linked with births from the same year.

### Covariates

Maternal race and ethnicity was self-reported on birth certificates and included American Indian or Alaska Native, Asian or Pacific Islander, Hispanic or Latinx, multiracial or other (multiracial and those self-identifying as other when asked about their race and ethnicity), non-Hispanic Black (Black), and non-Hispanic White (White) categories. Multiracial individuals were those who self-identified with at least 2 racial groups. In our models, we controlled for the following individual-level covariates considered to be significant risk factors of SMM: maternal age (<20, 20-34, or ≥35 years), maternal education (high school or less, some college, or college graduate), and insurance (private, public or government, other, unknown, or uninsured).^58^ On the basis of prior literature documenting relationships between area-level socioeconomic status, incarceration rates, and adverse birth outcomes, we also included county-level median household income with counties categorized into quartiles in our fully adjusted models.^48^ County-level median household income information was determined from the 2000 Decennial Census (for births between 1997 and 2004), the 2010 ACS 5-year estimates (for births between 2005 and 2010), the 2015 ACS 5-year estimates (for births between 2011 and 2015), and the 2019 ACS 5-year estimates (for births between 2016 and 2018) obtained from Social Explorer.^59^

### Statistical Analysis

Data analysis was performed from January 2022 to February 2023. In descriptive analyses, we determined the distribution of population characteristics by race and ethnicity and by tertiles of Black-White jail incarceration inequity. We then used race and ethnicity–stratified mixed-effects logistic regression models with random intercepts for counties to estimate odds ratios (ORs) and 95% CIs of NT SMM and SMM associated with county-level jail incarceration inequity comparing Black and White individuals. Our initial models were unadjusted (model 1). Our partially adjusted models included individual-level covariates (maternal age, education, and insurance) (model 2) and the fully adjusted models additionally controlled for county-level socioeconomic status (model 3). In sensitivity analysis, we added year fixed effects to our fully adjusted models to account for potential temporal trends (exogenous factors that change yearly and affect all counties). All hypothesis tests were 2-sided. All analyses were conducted using Stata/IC, version 15.1 (StataCorp LLC).

## Results

Our sample of 10 200 692 live births comprised 0.4% American Indian or Alaska Native, 13.4% Asian or Pacific Islander, 5.8% Black, 50.8% Hispanic or Latinx, 29.6% White, and 0.1% multiracial or other individuals. Black birthing people had the highest prevalence of SMM (0.9% NT SMM and 1.8% SMM), and White birthing people had the lowest prevalence (0.5% NT SMM and 1.0% SMM) (Table 1). Most birthing people resided in counties with high jail incarceration inequity (tertile 3), which remained the same across racial and ethnic groups (Table 1 and Table 2). The prevalence of NT SMM was evenly distributed across tertiles of jail incarceration inequity. However, the prevalence of SMM was slightly higher among birthing people living in low-inequity counties (tertile 1; 1.3%), whereas those living in moderate- and high-inequity counties had a similar prevalence (1.1%).

**Table 1.** Distribution of Population Characteristics Across Race and Ethnicity, California, 1997-2018

	No. (%)					
	American Indian or Alaska Native	Asian or Pacific Islander	Black	Hispanic or Latinx	White	Multiracial or other^a^
Births	43 254 (0.4)	1 369 170 (13.4)	589 692 (5.8)	5 177 325 (50.8)	3 014 652 (29.6)	6599 (0.1)
Non–blood transfusion severe maternal morbidity						
Yes	264 (0.6)	7678 (0.6)	5157 (0.9)	26 581 (0.5)	14 044 (0.5)	46 (0.7)
No	42 990 (99.4)	1 361 492 (99.4)	584 535 (99.1)	5 150 744 (99.5)	3 000 608 (99.5)	6553 (99.3)
Severe maternal morbidity^b^						
Yes	640 (1.5)	16 891 (1.2)	10 543 (1.8)	61 582 (1.2)	28 806 (1.0)	99 (1.5)
No	42 614 (98.5)	1 352 279 (98.8)	579 149 (98.2)	5 115 743 (98.8)	2 985 846 (99.0)	6500 (98.5)
Black-White jail incarceration inequity						
Tertile 1 (low inequity)	8916 (20.6)	65 911 (4.8)	73 207 (12.4)	685 081 (13.2)	387 932 (12.9)	597 (9.0)
Tertile 2	16 685 (38.6)	347 916 (25.4)	144 079 (24.4)	1 655 716 (32.0)	1 114 087 (37.0)	1432 (21.7)
Tertile 3 (high inequity)	17 653 (40.8)	955 343 (69.8)	372 406 (63.2)	2 836 528 (54.8)	1 512 633 (50.2)	4570 (69.3)
Maternal age, y						
<20	5383 (12.4)	29 900 (2.2)	74 500 (12.6)	618 056 (11.9)	130 249 (4.3)	388 (5.9)
20-34	32 449 (75.0)	973 004 (71.1)	432 315 (73.3)	3 883 014 (75.0)	2 182 370 (72.4)	4867 (73.8)
≥35	5422 (12.5)	366 266 (26.8)	82 877 (14.1)	676 255 (13.1)	702 033 (23.3)	1344 (20.4)
Maternal education						
High school or less	26 245 (60.7)	304 505 (22.2)	306 944 (52.1)	3 767 220 (72.8)	877 714 (29.1)	2676 (40.6)
Some college	12 137 (28.1)	304 578 (22.2)	192 521 (32.6)	987 989 (19.1)	820 119 (27.2)	1908 (28.9)
College graduate	4872 (11.3)	760 087 (55.5)	90 227 (15.3)	422 116 (8.2)	1 316 819 (43.7)	2015 (30.5)
Insurance						
Private	17 257 (39.9)	957 987 (70.0)	244 010 (41.4)	1 661 956 (32.1)	2 261 769 (75.0)	3368 (51.0)
Public or government	24 891 (57.5)	305 621 (22.3)	331 326 (56.2)	3 373 326 (65.2)	695 541 (23.1)	3067 (46.5)
Other, unknown, or uninsured	1106 (2.6)	105 562 (7.7)	14 356 (2.4)	142 043 (2.7)	57 342 (1.9)	164 (2.5)
County median household income						
Quartile 1 (low)	9359 (21.6)	27 168 (2.0)	13 835 (2.3)	317 698 (6.1)	214 731 (7.1)	273 (4.1)
Quartile 2	7223 (16.7)	63 041 (4.6)	37 388 (6.3)	491 999 (9.5)	283 511 (9.4)	388 (5.9)
Quartile 3	19 126 (44.2)	623 562 (45.5)	404 621 (68.6)	3 216 842 (62.1)	1 500 892 (49.8)	4155 (63.0)
Quartile 4 (high)	7546 (17.4)	655 399 (47.9)	133 848 (22.7)	1 150 786 (22.2)	1 015 518 (33.7)	1783 (27.0)

^a^ Multiracial or other category includes individuals who self-identified with at least 2 or more racial groups and those who self-identified as other race or ethnicity.

^b^ Severe maternal morbidity, including cases with blood transfusion as their only indicator.

**Table 2.** Distribution of Population Characteristics Across Tertiles of Black-White Inequity in County Jail Incarceration Rates, California, 1997-2018

	Black-White jail incarceration inequity, No. (%)		
	Tertile 1 (low inequity)	Tertile 2	Tertile 3 (high inequity)
Births	1 221 644 (12.0)	3 279 915 (32.2)	5 699 133 (55.9)
Non–blood transfusion severe maternal morbidity			
Yes	6164 (0.5)	16 551 (0.5)	31 055 (0.5)
No	1 215 480 (99.5)	3 263 364 (99.5)	5 668 078 (99.5)
Severe maternal morbidity^a^			
Yes	15 442 (1.3)	37 587 (1.1)	65 532 (1.1)
No	1 206 202 (98.7)	3 242 328 (98.9)	5 633 601 (98.9)
Maternal race and ethnicity			
American Indian or Alaska Native	8916 (0.7)	16 685 (0.5)	17 653 (0.3)
Asian or Pacific Islander	65 911 (5.4)	347 916 (10.6)	955 343 (16.8)
Black	73 207 (6.0)	144 079 (4.4)	372 406 (6.5)
Hispanic or Latinx	685 081 (56.1)	1 655 716 (50.5)	2 836 528 (49.8)
White	387 932 (31.8)	1 114 087 (34.0)	1 512 633 (26.5)
Multiracial or other^b^	597 (<0.1)	1432 (<0.1)	4570 (0.1)
Maternal age, y			
<20	138 884 (11.4)	290 364 (8.9)	429 228 (7.5)
20-34	930 849 (76.2)	2 461 705 (75.1)	4 115 465 (72.2)
≥35	151 911 (12.4)	527 846 (16.1)	1 154 440 (20.3)
Maternal education			
High school or less	741 723 (60.7)	1 761 240 (53.7)	2 782 341 (48.8)
Some college	304 018 (24.9)	793 697 (24.2)	1 221 537 (21.4)
College graduate	175 903 (14.4)	724 978 (22.1)	1 695 255 (29.7)
Insurance			
Private	516 743 (42.3)	1 633 885 (49.8)	2 995 719 (52.6)
Public or government	675 345 (55.3)	1 562 629 (47.6)	2 495 798 (43.8)
Other, unknown, or uninsured	29 556 (2.4)	83 401 (2.5)	207 616 (3.6)
County median household income			
Quartile 1 (low)	175 615 (14.4)	261 593 (8.0)	145 856 (2.6)
Quartile 2	305 598 (25.0)	400 182 (12.2)	177 770 (3.1)
Quartile 3	685 945 (56.1)	1 481 251 (45.2)	3 602 002 (63.2)
Quartile 4 (high)	54 486 (4.5)	1 136 889 (34.7)	1 773 505 (31.1)

^a^ Severe maternal morbidity, including cases with blood transfusion as their only indicator.

^b^ Multiracial or other category includes individuals who self-identified with at least 2 or more racial groups and those who self-identified as “other” race or ethnicity.

In models adjusting for individual sociodemographic characteristics, the odds of NT SMM for Black birthing people residing in counties with high Black-White jail incarceration inequity were 28% higher than those residing in low-inequity counties (OR, 1.28; 95% CI, 1.08-1.50) (model 2) (Table 3). This association remained consistent when examining SMM including blood transfusion–only cases (OR, 1.27; 95% CI, 1.07-1.49) (model 2) (Table 4). Associations were slightly attenuated when adjusting for county-level socioeconomic status (NT SMM: OR, 1.14; 95% CI, 1.01-1.29; SMM; OR, 1.20; 95% CI, 1.01-1.42) (model 3) (Table 3 and Table 4). Increased odds of NT SMM associated with residing in high-inequity counties among Black birthing people remained unchanged in our sensitivity analyses, where we additionally included year fixed effects to account for temporal trends (OR, 1.14; 95% CI, 1.01-1.29) (eTable 1 in Supplement 1). However, sensitivity analysis results were attenuated and no longer statistically significant for SMM (eTable 1 in Supplement 1).

**Table 3.** Associations Between Black-White Inequity in County Jail Incarceration Rates and Non–Blood Transfusion Severe Maternal Morbidity, California, 1997-2018

	Odds ratio (95% CI)		
	Model 1^a^	Model 2^b^	Model 3^c^
**American Indian or Alaska Native (n = 43 254)**			
Tertile 1 (low inequity)	1 [Reference]	1 [Reference]	1 [Reference]
Tertile 2	0.99 (0.67-1.45)	0.97 (0.66-1.41)	0.90 (0.61-1.32)
Tertile 3 (high inequity)	1.11 (0.76-1.62)	1.06 (0.73-1.54)	0.97 (0.66-1.43)
**Asian or Pacific Islander (n = 1 369 170)**			
Tertile 1 (low inequity)	1 [Reference]	1 [Reference]	1 [Reference]
Tertile 2	0.99 (0.84-1.18)	0.98 (0.83-1.16)	0.94 (0.79-1.11)
Tertile 3 (high inequity)	1.02 (0.86-1.22)	1.00 (0.85-1.19)	0.95 (0.80-1.13)
**Black (n = 589 692)**			
Tertile 1 (low inequity)	1 [Reference]	1 [Reference]	1 [Reference]
Tertile 2	1.20 (1.01-1.42)	1.19 (1.00-1.41)	1.08 (0.95-1.23)
Tertile 3 (high inequity)	1.30 (1.10-1.54)	1.28 (1.08-1.50)	1.14 (1.01-1.29)
**Hispanic or Latinx (n = 5 177 325)**			
Tertile 1 (low inequity)	1 [Reference]	1 [Reference]	1 [Reference]
Tertile 2	1.15 (1.07-1.23)	1.14 (1.06-1.23)	1.14 (1.06-1.22)
Tertile 3 (high inequity)	1.26 (1.16-1.36)	1.24 (1.15-1.34)	1.24 (1.14-1.34)
**White (n = 3 014 652)**			
Tertile 1 (low inequity)	1 [Reference]	1 [Reference]	1 [Reference]
Tertile 2	1.05 (0.97-1.13)	1.03 (0.95-1.12)	1.03 (0.94-1.12)
Tertile 3 (high inequity)	1.05 (0.97-1.14)	1.03 (0.94-1.12)	1.02 (0.93-1.12)
**Multiracial or other (n = 6599)** ^d^			
Tertile 1 (low inequity)	1 [Reference]	1 [Reference]	1 [Reference]
Tertile 2	1.14 (0.34-3.75)	1.2 (0.38-3.78)	1.22 (0.38-3.87)
Tertile 3 (high inequity)	1.00 (0.33-3.00)	1.08 (0.38-3.09)	0.96 (0.33-2.79)

^a^ Model 1 was unadjusted.

^b^ Model 2 was adjusted for maternal age, education, and insurance.

^c^ Model 3 was adjusted for maternal age, education, insurance, and county-level median household income.

^d^ Multiracial or other category includes individuals who self-identified with at least 2 or more racial groups and those who self-identified as “other” race or ethnicity.

**Table 4.** Associations Between Black-White Inequity in County Jail Incarceration Rates and Severe Maternal Morbidity (Including Blood Transfusion–Only Cases), California, 1997-2018

Characteristic	Odds ratio (95% CI)		
	Model 1^a^	Model 2^b^	Model 3^c^
**American Indian or Alaska Native (n = 43 254)**			
Tertile 1 (low inequity)	1 [Reference]	1 [Reference]	1 [Reference]
Tertile 2	0.88 (0.69-1.12)	0.88 (0.69-1.13)	0.86 (0.67-1.09)
Tertile 3 (high inequity)	0.93 (0.72-1.21)	0.93 (0.71-1.21)	0.88 (0.67-1.15)
**Asian or Pacific Islander (n = 1 369 170)**			
Tertile 1 (low inequity)	1 [Reference]	1 [Reference]	1 [Reference]
Tertile 2	1.01 (0.89-1.15)	1.00 (0.88-1.14)	1.01 (0.88-1.15)
Tertile 3 (high inequity)	1.12 (0.98-1.28)	1.10 (0.96-1.26)	1.11 (0.96-1.27)
**Black (n = 589 692)**			
Tertile 1 (low inequity)	1 [Reference]	1 [Reference]	1 [Reference]
Tertile 2	1.21 (1.04-1.41)	1.21 (1.04-1.41)	1.15 (0.99-1.35)
Tertile 3 (high inequity)	1.27 (1.08-1.50)	1.27 (1.07-1.49)	1.20 (1.01-1.42)
**Hispanic or Latinx (n = 5 177 325)**			
Tertile 1 (low inequity)	1 [Reference]	1 [Reference]	1 [Reference]
Tertile 2	1.12 (1.06-1.17)	1.11 (1.06-1.17)	1.11 (1.06-1.16)
Tertile 3 (high inequity)	1.21 (1.15-1.28)	1.20 (1.14-1.27)	1.20 (1.14-1.27)
**White (n = 3 014 652)**			
Tertile 1 (low inequity)	1 [Reference]	1 [Reference]	1 [Reference]
Tertile 2	1.07 (1.01-1.14)	1.07 (1.01-1.14)	1.08 (1.01-1.15)
Tertile 3 (high inequity)	1.09 (1.02-1.17)	1.08 (1.02-1.16)	1.09 (1.02-1.17)
**Multiracial or other (n = 6599)** ^d^			
Tertile 1 (low inequity)	1 [Reference]	1 [Reference]	1 [Reference]
Tertile 2	0.83 (0.39-1.79)	0.84 (0.39-1.81)	0.83 (0.38-1.79)
Tertile 3 (high inequity)	0.90 (0.46-1.76)	0.94 (0.48-1.83)	0.87 (0.43-1.75)

^a^ Model 1 was unadjusted.

^b^ Model 2 was adjusted for maternal age, education, and insurance.

^c^ Model 3 was adjusted for maternal age, education, insurance, and county-level median household income.

^d^ Multiracial or other category includes individuals who self-identified with at least 2 or more racial groups and those who self-identified as “other” race or ethnicity.

Results were generally similar for Hispanic and Latinx birthing people. After adjustment for individual characteristics and county-level socioeconomic status, the odds of NT SMM were 24% higher and the odds of SMM were 20% higher for those residing in high-inequity counties (NT SMM: OR, 1.24, 95% CI, 1.14-1.34; SMM: OR, 1.20; 95% CI, 1.14-1.27) (Table 3 and Table 4). In sensitivity analyses, the association observed for NT SMM was moderately attenuated when including year fixed effects (OR, 1.09; 95% CI, 1.01-1.18), whereas the association observed for SMM was no longer significant (eTable 1 in Supplement 1). For Black and Hispanic or Latinx birthing people, associations comparing moderate- vs low-tertile counties were more attenuated across both SMM specifications (Table 3 and Table 4) but did not remain precise when adjusting for temporal trends (eTable 1 in Supplement 1).

Among White birthing people, we observed higher odds of SMM associated with residing in high-tertile counties only when considering SMM including blood transfusion–only cases (NT SMM: OR, 1.02; 95% CI, 0.93-1.12; SMM: OR, 1.09; 95% CI, 1.02-1.17) (model 3) (Table 3 and Table 4). However, this association was not significant when controlling for temporal trends (eTable 1 in Supplement 1). Estimates for American Indian or Alaska Native (NT SMM: OR, 0.97; 95% CI, 0.66-1.43; SMM: OR, 0.88; 95% CI, 0.67-1.15), Asian or Pacific Islander (NT SMM: OR, 0.95; 95% CI, 0.80-1.13; SMM: OR, 1.11; 95% CI, 0.96-1.27), and multiracial or other (NT SMM: OR, 0.96; 95% CI, 0.33-2.79; SMM: OR, 0.87; 95% CI, 0.43-1.75) individuals residing in counties with the highest Black-White jail incarceration inequity were null and imprecise (Table 3 and Table 4). Full regression results are presented in eTables 2 and 3 in Supplement 1.

## Discussion

In this study, we leveraged statewide data from California over 21 years and examined how county jail incarceration inequity between Black and White individuals may be related to SMM risk and its racial and ethnic disparities. Consistent with our hypothesis, we found that for Black and Hispanic or Latinx birthing people, residing in counties with greater jail incarceration inequity was associated with increased risk of NT SMM and SMM compared with residing in low-inequity counties. Associations between county-level jail incarceration inequity and NT SMM were stronger for Black individuals, even when accounting for temporal trends. Our results underscore the multilevel harmful consequences of the criminal-legal system as one key domain of structural racism and highlight the urgent need for structural transformation.

A recent systematic review identified only 6 epidemiologic studies assessing how structural racism indicators shaped maternal morbidity and mortality and found that most studies used either a measure of residential segregation or spatial isolation to operationalize structural racism.^60^ Only 1 study examined Black-White incarceration inequity as a measure of structural racism and found no association between this county-level indicator and SMM.^50^ Since that review, 1 other study has investigated racial inequity in incarceration within counties of the hospitals where individuals gave birth and did not find an association with SMM.^51^ To our knowledge, our study is the first to document associations between county-level jail incarceration inequity and increased SMM risk. These results align with prior studies that have found that living in areas with greater Black-White inequity in incarceration rates is associated with small for gestational age births,^47^ low birth weight,^48^ preterm birth,^43^ and infant mortality.^49^ Taken together, our findings contribute to the limited but growing body of empirical literature on the links between indicators of structural racism and birthing outcomes more broadly, particularly through the criminal-legal system.

The results of our study are also aligned with theoretical framings that outline pathways by which structural racism operating via the criminal-legal system may shape adverse health outcomes. Inequities in incarceration rates indicate systematic disinvestment in low-income and racially marginalized communities.^25,33^ Therefore, such areas may have poor neighborhood physical and social environment attributes that influence health-related behaviors, chronic stress, stress-buffering resources, and access to quality health care, which consequently determine the risk of adverse pregnancy-related outcomes.^13,61,62,63,64^ Inequities in incarceration rates also correspond to heightened state surveillance within Black and other racially and ethnically marginalized communities.^31,33^ Chronic stress arising from fear and worry about arrest and incarceration can be biologically embodied to disrupt a wide range of physiologic systems.^65,66,67,68^ Heightened surveillance also indicates resources diverted away from essential health and social services and instead toward policing these same communities.^25,28,67^ Hence, by stripping marginalized communities of stable social and financial resources essential for healthy pregnancies and childbirth, mass incarceration drives inequities in maternal health.^36,37^ These collateral consequences are all in addition to the economic, emotional, social, and health harms that incarcerated individuals and their families sustain, both during imprisonment and after being released, due to exclusion from access to public benefits, employment, and political activity.^24,33^

Our study also provides a unique understanding of the population-level impact of mass incarceration and structural racism, specifically within California. California incarcerates a higher percentage of its population than entire nations, such as the UK, and is home to significant racial inequities.^29^ In California, jail incarceration rates are 3 to 4 times higher for Black individuals than White individuals.^69^ Data also indicate that although Black individuals comprise only 6% of the state’s population, they represent 20% and 28% of the state’s jail and prison populations, respectively.^69,70^ This finding illustrates that Black individuals are likely to know someone incarcerated in their social networks or community. Estimates indicate that in the US, 44% of Black women have an incarcerated family member compared with 12% of White women.^71^ This large difference points to strained emotional, instrumental, and financial resources and increased psychosocial and economic stressors within Black and other racially and ethnically marginalized communities due to a family member, a loved one, a neighbor, or a community member being incarcerated.^72^ This, combined with other stressors that disproportionately burden racially and ethnically minoritized individuals, differentially heightens the risk of adverse health outcomes. As such, our study shows that racial inequities in incarceration within California also translate to racial inequities in adverse birthing outcomes. Notably, although jail incarceration inequity was associated with increased risk of SMM for Black, Hispanic or Latinx, and White birthing people, associations were stronger for Black birthing people, particularly when examining NT SMM, even when accounting for county socioeconomic status and temporal trends.

### Limitations

This study has several limitations. First, due to a lack of data on incarceration rates at different geographic levels, we assessed exposure at the county level. Counties are large geographic entities and likely contain heterogeneous exposure distribution within smaller subgeographies. This highlights the need for local, state, and federal agencies to make data available at more granular geographic levels for population health research. On the other hand, counties may be more conducive to policy-level interventions that may alleviate inequities due to their governance structure.^73^ Second, structural racism is a multidimensional construct that manifests through several social and cultural institutions.^74^ However, our study only assesses its impact through the criminal-legal system and does not capture how other domains of structural racism may contribute to the risk of SMM. Hence, future studies should implement multidimensional approaches to better estimate the influence of structural racism on population health outcomes through its multiple domains.^75^ Furthermore, this study does not explicitly address policies that shape incarceration rates or racial disparities in sentencing practices, which are also important dimensions of structural racism.^76^ Particularly, within the context of California, we did not examine how laws such as Public Safety Realignment, which shifted jurisdiction of certain criminal cases from the state to counties, may be associated with racial and ethnic inequities in jail incarceration rates.^77^ Future studies should examine how structural racism influences adverse health outcomes via different policies and practices. Third, although we controlled for a range of individual-level characteristics and county socioeconomic status, we cannot completely rule out the possibility of social selection influencing our results.^78^

## Conclusions

Structural racism, entrenched within several social and cultural domains in the US, has far-reaching consequences and drives racial and ethnic health inequities. One such domain is the criminal-legal system, which incarcerates Black and other racially and ethnically marginalized individuals at much higher rates than White individuals. In this study, we found that residing in counties with high jail incarceration inequity between Black and White individuals was associated with increased SMM risk, particularly among Black and Hispanic or Latinx birthing people.

## Notes

Supplement 1. **eFigure.** Study Sample Inclusion Flowchart **eTable 1.** Associations Between Black-White Inequity in County Jail Incarceration Rates and Severe Maternal Morbidity Accounting for Temporal Trends, California 1997-2018 **eTable 2.** Associations Between Black-White Inequity in County Jail Incarceration Rates and Non-Blood Transfusion Severe Maternal Morbidity, California 1997-2018 **eTable 3.** Associations Between Black-White Inequity in County Jail Incarceration Rates and Severe Maternal Morbidity (Including Blood Transfusion–Only Cases), California 1997-2018

Supplement 2.
Data Sharing Statement
