# Supplementary material for: Structural Racism, Mass Incarceration, and Racial and Ethnic Disparities in Severe Maternal Morbidity
Source: JAMA Netw Open. 2024 Jan 26;7(1):e2353626. doi: 10.1001/jamanetworkopen.2023.53626 (PMC10818215; doi:10.1001/jamanetworkopen.2023.53626)
Supplement: Supplement 1. — eFigure. Study Sample Inclusion Flowchart eTable 1. Associations Between Black-White Inequity in County Jail Incarceration Rates and Severe Maternal Morbidity Accounting for Temporal Trends, California 1997-2018 eTable 2. Associations Between Black-White Inequity in County Jail Incarceration Rates and Non-Blood Transfusion Severe Maternal Morbidity, California 1997-2018 eTable 3. Associations Between Black-White Inequity in County Jail Incarceration Rates and Severe Maternal Morbidity (Including Blood Transfusion–Only Cases), California 1997-2018 [file jamanetwopen-e2353626-s001.pdf]

Supplementary Online Content

Hailu EM, Riddell CA, Bradshaw PT, Ahern J, Carmichael SL, Mujahid MS. Structural racism, mass incarceration, and racial and ethnic disparities in severe maternal morbidity. *JAMA Netw Open*. 2024;7(1):e2353626. doi:10.1001/jamanetworkopen.2023.53626

eFigure. Study Sample Inclusion Flowchart

eTable 1. Associations Between Black-White Inequity in County Jail Incarceration Rates and Severe Maternal Morbidity Accounting for Temporal Trends, California 1997-2018

eTable 2. Associations Between Black-White Inequity in County Jail Incarceration Rates and Non-Blood Transfusion Severe Maternal Morbidity, California 1997-2018

eTable 3. Associations Between Black-White Inequity in County Jail Incarceration Rates and Severe Maternal Morbidity (including blood transfusion-only cases), California 1997-2018

This supplementary material has been provided by the authors to give readers additional information about their work.

© 2024 Hailu EM et al. *JAMA Network Open.*

eFigure 1: Study Sample Inclusion Flowchart

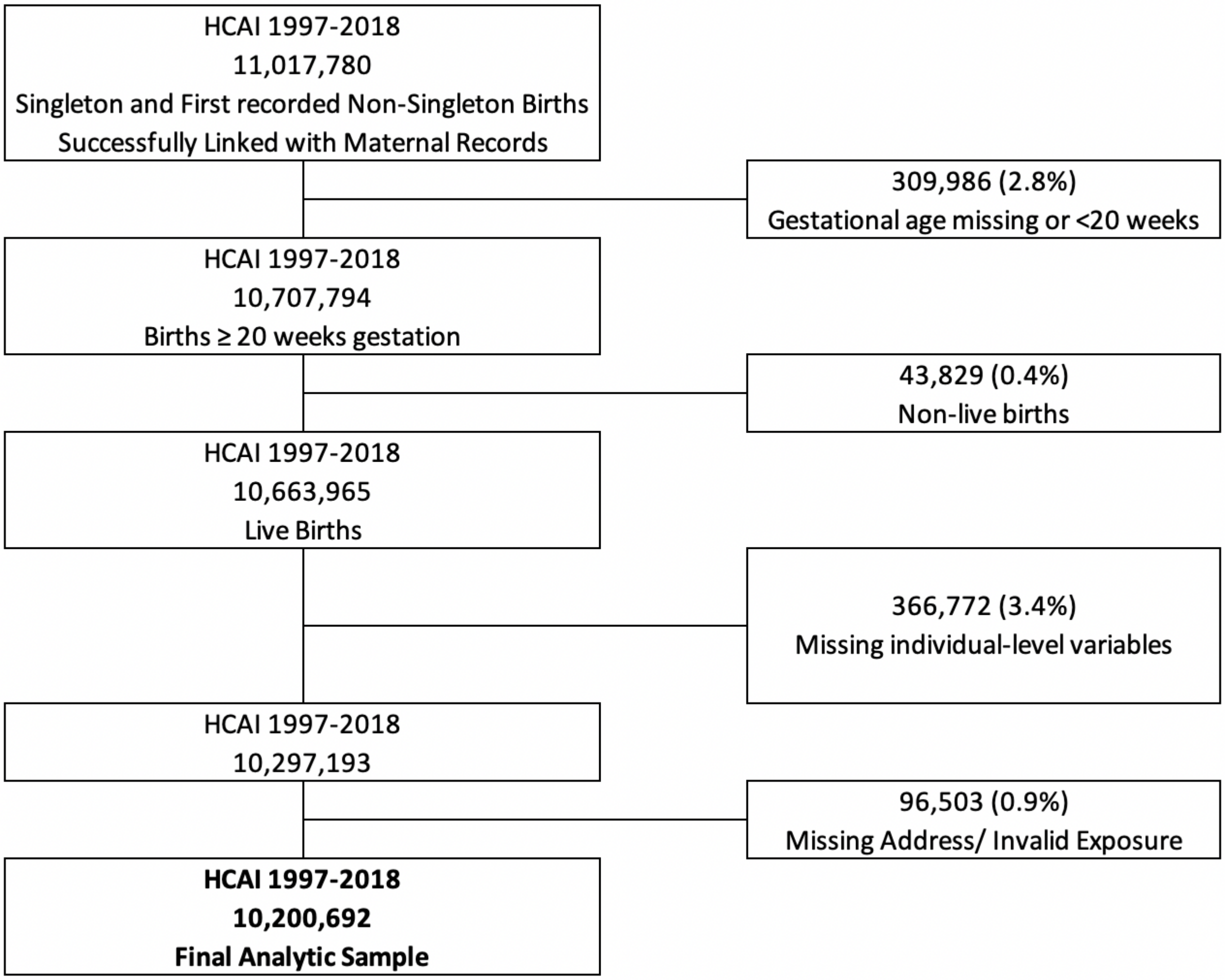

*HCAI: Department of Health Care Access and Information. HCAI linked vital statistics records with maternal birth hospitalization discharge records

© 2024 Hailu EM et al. *JAMA Network Open.*

eTable 1: Associations between Black-White Inequity in County Jail Incarceration Rates and Severe Maternal Morbidity Accounting for Temporal Trends, California 1997-2018.[a]

	American Indian or Alaska Native (N=43,254)	*Asian or Pacific Islander (N=1,369,170)*	*Black (N=589,692)*	*Hispanic or Latinx (N=5,177,325)*	*White (N=3,014,652)*	*Multiracial or other [b] (N=6,599)*
	OR (95% CI)	OR (95% CI)	OR (95% CI)	OR (95% CI)	OR (95% CI)	OR (95% CI)
**Non-blood transfusion severe maternal morbidity**						
Black-White Inequity in County Jail Incarceration Rates						
Tertile 1 (Low inequity)	1 [Reference]	1 [Reference]	1 [Reference]	1 [Reference]	1 [Reference]	NE [c]
Tertile 2	0.89 (0.60, 1.32)	0.94 (0.79, 1.11)	1.06 (0.94, 1.20)	1.05 (0.98, 1.13)	0.97 (0.89, 1.06)	NE
Tertile 3 (High inequity)	0.96 (0.65, 1.42)	0.87 (0.73, 1.04)	1.14 (1.01, 1.29)	1.09 (1.01, 1.18)	0.94 (0.86, 1.03)	NE
**Severe maternal morbidity (including blood transfusion-only cases)**						
Black-White Inequity in County Jail Incarceration Rates						
Tertile 1 (Low inequity)	1 [Reference]	1 [Reference]	1 [Reference]	1 [Reference]	1 [Reference]	1 [Reference]
Tertile 2	0.84 (0.66, 1.07)	0.95 (0.84, 1.09)	1.02 (0.88, 1.19)	0.96 (0.92, 1.01)	0.97 (0.91, 1.03)	0.81 (0.37, 1.76)
Tertile 3 (High inequity)	0.89 (0.68, 1.16)	0.94 (0.82, 1.07)	1.01 (0.86, 1.19)	0.99 (0.93, 1.04)	0.95 (0.89, 1.01)	0.88 (0.43, 1.77)

[a] Estimates are from mixed effects regression models with random intercepts for counties and year fixed effects, adjusting for maternal age, education, insurance, and county median household income

[b] Multiracial or other category includes individuals who self-identified with at least 2 or more racial groups and those who self-identified as “other” race/ethnicity

[c] NE=not estimable due to high instability given low sample sizes

© 2024 Hailu EM et al. *JAMA Network Open.*

eTable 2: Associations between Black-White Inequity in County Jail Incarceration Rates and Non-blood Transfusion Severe Maternal Morbidity, California 1997-2018. [a]

	American Indian or Alaska Native (N=43,254)	*Asian or Pacific Islander (N=1,369,170)*	*Black (N=589,692)*	*Hispanic or Latinx (N=5,177,325)*	*White (N=3,014,652)*	*Multiracial or other [b] (N=6,599)*
	OR (95% CI)	OR (95% CI)	OR (95% CI)	OR (95% CI)	OR (95% CI)	OR (95% CI)
Black-White Jail Incarceration Inequity						
Tertile 1 (Low inequity)	1 [Reference]	1 [Reference]	1 [Reference]	1 [Reference]	1 [Reference]	1 [Reference]
Tertile 2	0.90 (0.61, 1.32)	0.94 (0.79, 1.11)	1.08 (0.95, 1.23)	1.14 (1.06, 1.22)	1.03 (0.94, 1.12)	1.22 (0.38, 3.87)
Tertile 3 (High inequity)	0.97 (0.66, 1.43)	0.95 (0.80, 1.13)	1.14 (1.01, 1.29)	1.24 (1.14, 1.34)	1.02 (0.93, 1.12)	0.96 (0.33, 2.79)
Maternal age, y						
<20	1 [Reference]	1 [Reference]	1 [Reference]	1 [Reference]	1 [Reference]	1 [Reference]
20-34	0.84 (0.57, 1.25)	1.33 (1.10, 1.62)	1.24 (1.13, 1.37)	0.92 (0.88, 0.95)	1.16 (1.06, 1.28)	0.77 (0.26, 2.28)
≥35	1.78 (1.13, 2.82)	2.40 (1.97, 2.92)	2.10 (1.88, 2.35)	1.59 (1.52, 1.66)	1.81 (1.64, 1.99)	1.16 (0.34, 3.89)
Maternal education						
High school or less	0.95 (0.62, 1.43)	0.99 (0.93, 1.06)	1.00 (0.92, 1.09)	0.97 (0.93, 1.02)	1.00 (0.95, 1.04)	0.99 (0.43, 2.32)
Some college	1.09 (0.72, 1.64)	1.01 (0.95, 1.07)	1.00 (0.92, 1.09)	1.02 (0.97, 1.07)	1.02 (0.98, 1.07)	0.82 (0.34, 1.97)
College graduate	1 [Reference]	1 [Reference]	1 [Reference]	1 [Reference]	1 [Reference]	1 [Reference]
Insurance						
Private	1 [Reference]	1 [Reference]	1 [Reference]	1 [Reference]	1 [Reference]	1 [Reference]
Public/government	1.04 (0.79, 1.36)	1.07 (1.00, 1.13)	1.15 (1.08, 1.22)	1.05 (1.02, 1.08)	1.18 (1.13, 1.24)	2.06 (1.01, 4.22)
Other/unknown/uninsured	0.45 (0.14, 1.44)	0.72 (0.65, 0.79)	1.05 (0.87, 1.26)	0.94 (0.86, 1.02)	1.08 (0.96, 1.22)	1.2 (0.16, 9.22)
County Median Household Income						
Quartile 1 (Low)	0.95 (0.60, 1.49)	0.83 (0.66, 1.03)	0.71 (0.57, 0.89)	0.98 (0.86, 1.12)	0.93 (0.83, 1.04)	0.79 (0.09, 6.84)
Quartile 2	0.71 (0.42, 1.17)	0.76 (0.64, 0.91)	0.74 (0.63, 0.86)	0.97 (0.86, 1.10)	0.99 (0.88, 1.11)	0.58 (0.07, 5.04)
Quartile 3	1.06 (0.73, 1.54)	1.05 (0.92, 1.20)	0.91 (0.84, 1.00)	1.06 (0.94, 1.19)	1.10 (1.00, 1.22)	2.33 (0.96, 5.66)
Quartile 4 (High)	1 [Reference]	1 [Reference]	1 [Reference]	1 [Reference]	1 [Reference]	1 [Reference]

[a] Estimates are from mixed effects regression models with random intercepts for counties, adjusting for maternal age, education, insurance, and county median household income

[b] Multiracial or other category includes individuals who self-identified with at least 2 or more racial groups and those who self-identified as “other” race/ethnicity

© 2024 Hailu EM et al. *JAMA Network Open.*

eTable 3: Associations between Black-White Inequity in County Jail Incarceration Rates and Severe Maternal Morbidity (including blood transfusion-only cases), California 1997-2018.[a]

	American Indian or Alaska Native (N=43,254)	*Asian or Pacific Islander (N=1,369,170)*	*Black (N=589,692)*	*Hispanic or Latinx (N=5,177,325)*	*White (N=3,014,652)*	*Multiracial or other [b] (N=6,599)*
	OR (95% CI)	OR (95% CI)	OR (95% CI)	OR (95% CI)	OR (95% CI)	OR (95% CI)
Black-White Jail Incarceration Inequity						
Tertile 1 (Low inequity)	1 [Reference]	1 [Reference]	1 [Reference]	1 [Reference]	1 [Reference]	1 [Reference]
Tertile 2	0.86 (0.67, 1.09)	1.01 (0.88, 1.15)	1.15 (0.99, 1.35)	1.11 (1.06, 1.16)	1.08 (1.01, 1.15)	0.83 (0.38, 1.79)
Tertile 3 (High inequity)	0.88 (0.67, 1.15)	1.11 (0.96, 1.27)	1.20 (1.01, 1.42)	1.20 (1.14, 1.27)	1.09 (1.02, 1.17)	0.87 (0.43, 1.75)
Maternal age, y						
<20	1 [Reference]	1 [Reference]	1 [Reference]	1 [Reference]	1 [Reference]	1 [Reference]
20-34	0.98 (0.77, 1.26)	1.09 (0.97, 1.23)	1.22 (1.15, 1.31)	0.85 (0.83, 0.88)	1.07 (1.01, 1.14)	1.15 (0.49, 2.71)
≥35	1.78 (1.32, 2.41)	1.81 (1.61, 2.04)	2.11 (1.96, 2.28)	1.28 (1.24, 1.32)	1.59 (1.49, 1.70)	1.33 (0.51, 3.44)
Maternal education						
High school or less	1.12 (0.84, 1.50)	0.93 (0.89, 0.97)	1.05 (0.99, 1.12)	0.96 (0.93, 0.99)	1.01 (0.98, 1.04)	1.14 (0.65, 2.01)
Some college	1.17 (0.87, 1.57)	1.00 (0.97, 1.04)	1.04 (0.97, 1.10)	1.04 (1.01, 1.08)	1.01 (0.98, 1.04)	0.92 (0.51, 1.64)
College graduate	1 [Reference]	1 [Reference]	1 [Reference]	1 [Reference]	1 [Reference]	1 [Reference]
Insurance						
Private	1 [Reference]	1 [Reference]	1 [Reference]	1 [Reference]	1 [Reference]	1 [Reference]
Public/government	1.25 (1.04, 1.50)	1.16 (1.11, 1.20)	1.28 (1.23, 1.34)	1.17 (1.15, 1.19)	1.28 (1.24, 1.32)	1.5 (0.93, 2.41)
Other/unknown/uninsured	1.14 (0.68, 1.90)	0.91 (0.85, 0.97)	1.22 (1.08, 1.38)	1.08 (1.02, 1.14)	1.13 (1.04, 1.23)	1.5 (0.45, 4.95)
County Median Household Income						
Quartile 1 (Low)	1.07 (0.79, 1.45)	1.01 (0.82, 1.25)	0.97 (0.77, 1.22)	1.31 (1.12, 1.53)	1.06 (0.94, 1.18)	0.94 (0.31, 2.85)
Quartile 2	0.78 (0.55, 1.11)	1.03 (0.85, 1.25)	0.83 (0.67, 1.02)	1.21 (1.04, 1.40)	1.06 (0.95, 1.18)	0.67 (0.22, 2.03)
Quartile 3	0.95 (0.72, 1.25)	1.20 (1.01, 1.44)	1.06 (0.87, 1.28)	1.35 (1.18, 1.55)	1.19 (1.08, 1.31)	1.13 (0.69, 1.85)
Quartile 4 (High)	1 [Reference]	1 [Reference]	1 [Reference]	1 [Reference]	1 [Reference]	1 [Reference]

[a] Estimates are from mixed effects regression models with random intercepts for counties, adjusting for maternal age, education, insurance, and county median household income

[b] Multiracial or other category includes individuals who self-identified with at least 2 or more racial groups and those who self-identified as “other” race/ethnicity

© 2024 Hailu EM et al. *JAMA Network Open.*
