# Supplementary material for: Structural Racism, Mass Incarceration, and Racial and Ethnic Disparities in Severe Maternal Morbidity
Source: JAMA Netw Open. 2024 Jan 26;7(1):e2353626. doi: 10.1001/jamanetworkopen.2023.53626 (PMC10818215; doi:10.1001/jamanetworkopen.2023.53626)
Supplement: Supplement 2. — Data Sharing Statement [file jamanetwopen-e2353626-s002.pdf]

Data Sharing Statement

Hailu. Structural Racism, Mass Incarceration, and Racial and Ethnic Disparities in Severe Maternal Morbidity. *JAMA Netw Open*. Published January 26, 2024.
doi:10.1001/jamanetworkopen.2023.53626

Data
Data available: No
